# Combination of lymphocyte number and function in evaluating host immunity

**DOI:** 10.18632/aging.102595

**Published:** 2019-12-20

**Authors:** Ying Luo, Yalong Xie, Weijie Zhang, Qun Lin, Guoxing Tang, Shiji Wu, Min Huang, Botao Yin, Jin Huang, Wei Wei, Jing Yu, Hongyan Hou, Liyan Mao, Weiyong Liu, Feng Wang, Ziyong Sun

**Affiliations:** 1 Department of Laboratory Medicine, Tongji Hospital, Tongji Medical College, Huazhong University of Science and Technology, Wuhan, China; 2 Institute of Organ Transplantation, Tongji Hospital, Tongji Medical College, Huazhong University of Science and Technology, Wuhan, China

**Keywords:** lymphocyte number, lymphocyte function, host immunity, evaluation

## Abstract

Accurate monitoring of host immunity is hampered by the flaws of conventional tests. The relationship between lymphocyte number and function is unknown. The function of lymphocytes was analyzed based on IFN-γ secretion assay. Lymphocyte number and function was investigated in individuals under various states. The number of CD4^+^ and CD8^+^ T cells was gradually decreased, whereas the function of them was gradually increased with increasing age. A significantly negative correlation existed between the number and function of both CD4^+^ and CD8^+^ T cells. Differently, both the number and function of NK cells are maintained at a high level after birth. Staying up all night was found to impair the function of CD4^+^, CD8^+^ T cells, or NK cells. Lymphocyte number and function were both decreased in patients with immunosuppressive conditions or opportunistic infections, while the opposite phenomenon was observed in patients with some autoimmune diseases (except for NK cells). In kidney transplant recipients, the number and function of CD4^+^ and CD8^+^ T cells were increased or decreased when rejection or infection occurred. We demonstrated that evaluation of host immunity based on combination of lymphocyte number and function plays an important role in the diagnosis, treatment, and prognosis of diseases.

## INTRODUCTION

Lymphocyte responses remain one of the most important strategies to reflect immune function [1–9]. However, assessment and monitoring of lymphocyte function is hampered by the flaws of conventional tests. Although the current tests, including lymphocyte subset and intracellular cytokine analyses, are broadly used in clinical practice, they cannot truly reflect lymphocyte function [8, 10–15]. Thus, there is an urgent need for more precise methods to evaluate host immunity in clinical practice.

Lymphocytes (CD4^+^ T cells, CD8^+^ T cells, and NK cells) have multiple activities, such as proliferation, activation, and cytotoxicity. It is impossible to assess the whole activities of lymphocytes in clinical practice, since the procedures will be very complicated and time-consuming. Finding the markers which can be used as a symbol of lymphocyte activities is necessary. We have demonstrated that the production of interferon-gamma (IFN-γ) in CD4^+^ T cells, CD8^+^ T cells, and NK cells under phorbol-12-myristate-13-acetate/Ionomycin (PMA/Ionomycin) stimulation was well correlated with the activation, chemotaxis, and cytotoxicity of them, which suggests that IFN-γ producing capability can be used as a marker of lymphocyte function [16]. This method is simple (no need to purify cells, using one stimulant, and simultaneously detecting the function of CD4^+^ T cells, CD8^+^ T cells, and NK cells), rapid (4 hours of stimulation plus 2 hours of antibody labeling), and safe (no hazards of radiation), thus it is of great value in clinical practice.

There are some studies focusing on the number of lymphocytes in different ages or different diseases. However, the function of lymphocytes in these conditions is rarely reported because of lack of standardized method. There is no study to determine the relationship between lymphocyte number and function in humans at different age stages. In this study, we first reported that CD4^+^ and CD8^+^ T cell number is negatively correlated with their function with increasing age. Our data suggest that the impairment of immunity in children is caused by dysfunction of CD4^+^ and CD8^+^ T cells, while the impairment of that in old people is caused by insufficient number of them.

## RESULTS

### The optimization and validation of PMA/Ionomycin-stimulated lymphocyte function assay

We have made some optimizations for PMA/Ionomycin-stimulated lymphocyte function assay. V450-labeled anti-CD4 was replaced by allophycocyanin Cy7 (APC-Cy7), and phycoerythrin (PE)-labeled anti-IFN-γ was replaced by APC, as the latter two antibodies had better performance in distinguishing between positive and negative cells, especially for CD4^+^ and CD8^+^ T cells (Supplementary Figure 1A, 1B). The antibody information used in this research is shown in Supplementary Table 1.

**Figure 1:**
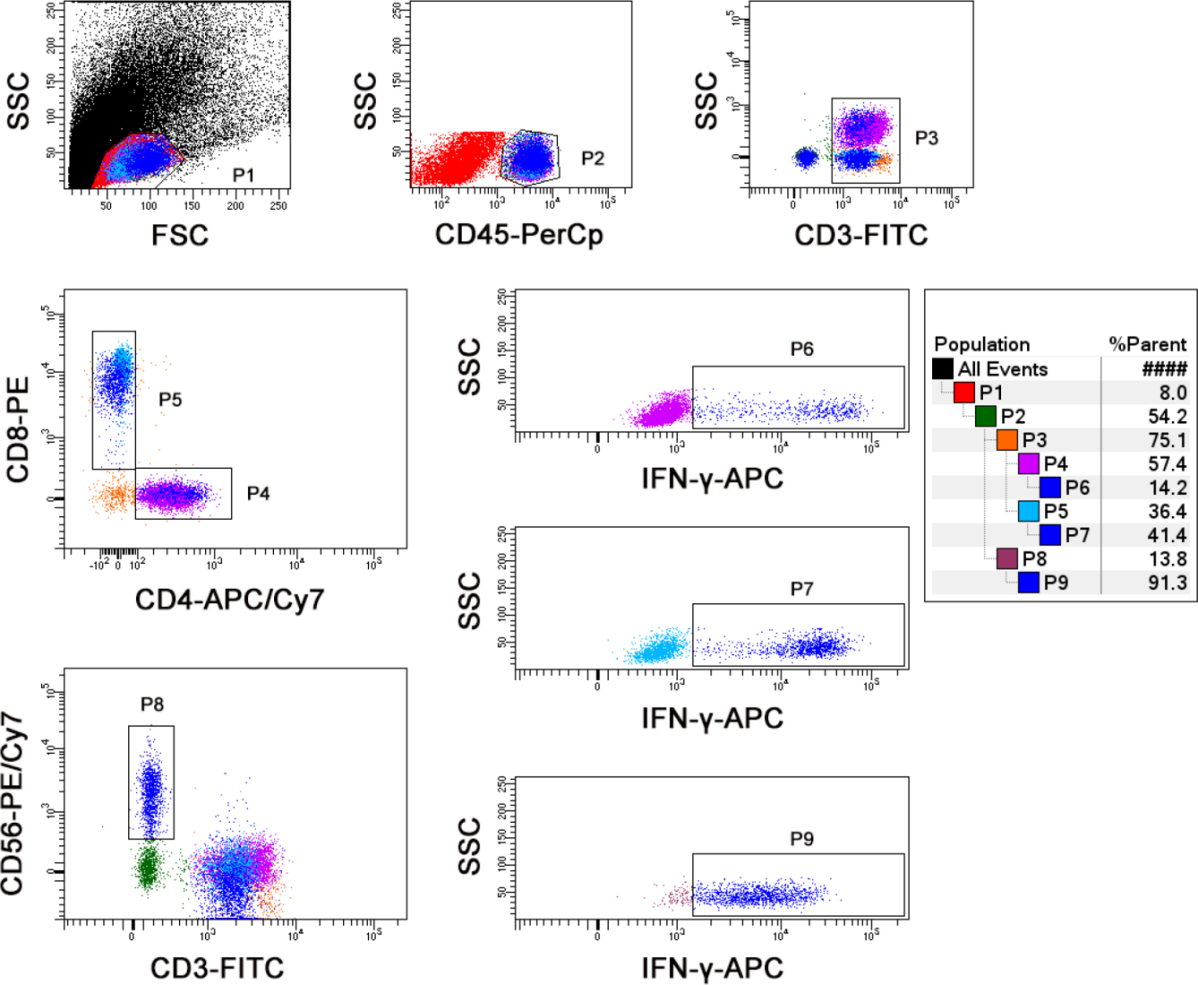
**The final flow analysis template of lymphocyte function assay.** Diluted whole blood was stimulated with Leukocyte Activation Cocktail for 4 h. Representative flow plots showing the gating strategies of IFN-γ^+^ cells in CD4^+^, CD8^+^ T cells, and NK cells.

Using different batches of reagents did not affect the results of lymphocyte function assay (Supplementary Figure 2A, Supplementary Table 2). The intra-assay repeatability was evaluated by analyzing 20 replicates and coefficients of variations (CVs) were 3.26% for CD4^+^ T cells, 2.75% for CD8^+^ T cells, and 3.23% for NK cells, respectively (Supplementary Figure 2B). Lymphocyte function was continuously detected (4-7 times) in 4 healthy individuals within 2 months and the CVs for CD4^+^ T cells, CD8^+^ T cells, and NK cells were all less than 13% (Supplementary Figure 2C). No significant changes were observed in the function of CD4^+^ and CD8^+^ T cells for samples within 48 h. However, NK cell function was significantly decreased after 24 h of sample collection (Supplementary Figure 2D). Finally, we have established the standard protocol for PMA/Ionomycin-stimulated lymphocyte function assay, and the flow analysis template is shown in Figure 1.

### The number and function of lymphocytes in different age stages

**Figure 2:**
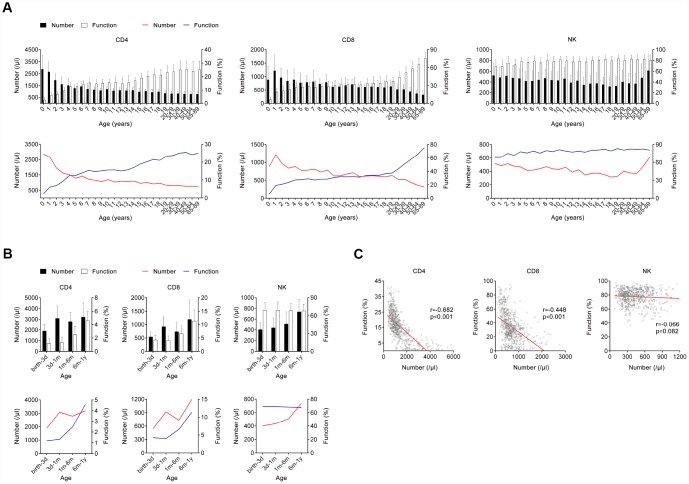
**The number and function of lymphocytes in different age stages of healthy individuals.** (**A**) Bar graphs showing the number and function of CD4^+^, CD8^+^ T cells, and NK cells in healthy individuals at different ages (between 0 and 89 years). Data are shown as means ± SD. Line diagrams showing the mean values of number and function of CD4^+^, CD8^+^ T cells, or NK cells at different ages (between 0 and 89 years). (**B**) Bar graphs showing the number and function of CD4^+^, CD8^+^ T cells, and NK cells in infants at different ages (between birth and 12 months). Data are shown as means ± SD. Line diagrams showing the mean values of number and function of CD4^+^, CD8^+^ T cells, or NK cells at different ages (between birth and 12 months). (**C**) Correlation between the number and function of CD4^+^, CD8^+^ T cells, and NK cells in all participants. Each symbol represents an individual donor.

**Figure 3:**
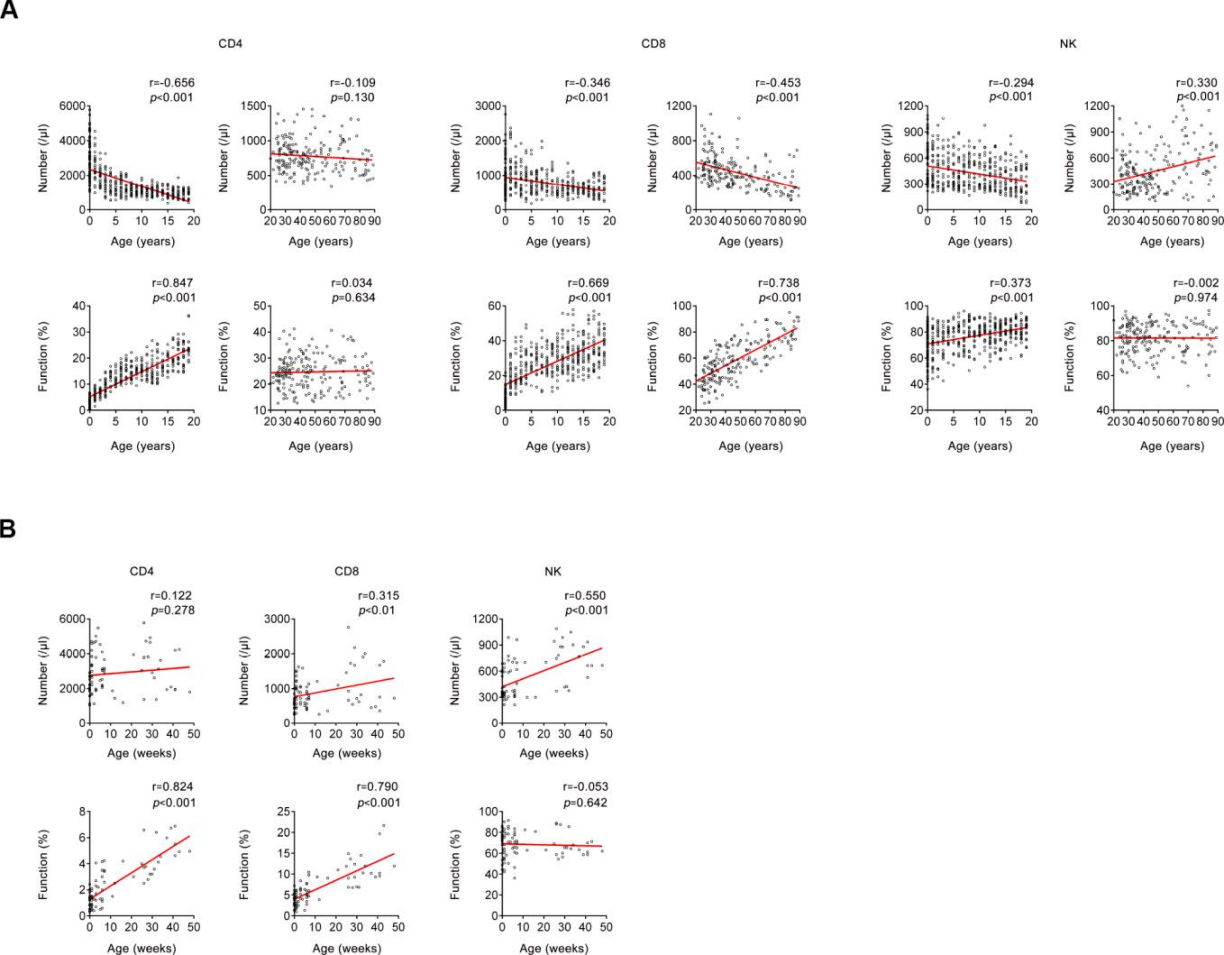
**Correlation between host immunity and age.** (**A**) Correlation between host immunity (the number and function of CD4^+^, CD8^+^ T cells, and NK cells) and age (between 0 and 20 years; > 20 years). (**B**) Correlation between host immunity (the number and function of CD4^+^, CD8^+^ T cells, and NK cells) and age (between 0 and 50 weeks). Each symbol represents an individual donor.

A total of 695 (male, 49.64%) healthy participants with different ages were recruited. Generally, the number of CD4^+^ and CD8^+^ T cells was gradually decreased but the function of them was gradually increased with growing age, while both the number and function of NK cells were maintained at a high level after birth (Figure 2A). Further correlation analysis showed that the number and function of CD4^+^ T cells was negatively and positively correlated with age, respectively, but this occurred only among people younger than 20 years of age. The same phenomenon was found in CD8^+^ T cells during lifetime. However, this phenomenon was not obviously observed in NK cells (Figure 3A).

We also divided infants aged < 1 year old into different month groups, since the host immunity was very special during that period. Both the number and function of CD4^+^ and CD8^+^ T cells were gradually increased with growing months. Differently, only the number of NK cells was gradually increased with growing months, while the function of them did not change during that period (Figures 2B, 3B).

For the number of CD4^+^ and CD8^+^ T cells, no significant difference was found between male and female in all age groups. However, for the number of NK cells, there was a significant difference between male and female over the age of 20 years. No significant difference was found between male and female in all age groups for the function of CD4^+^, CD8^+^ T cells, and NK cells (Supplementary Table 3). The intervals of lymphocyte number and function in different ages are shown in Supplementary Table 3.

We further analyzed the correlation between the number and function of lymphocytes in all participants. Interestingly, there was a significant negative correlation between the number and function of CD4^+^ and CD8^+^ T cells, but no significant correlation was found between the number and function of NK cells (Figure 2C).

### The effect of different risk factors on the number and function of lymphocytes

**Figure 4:**
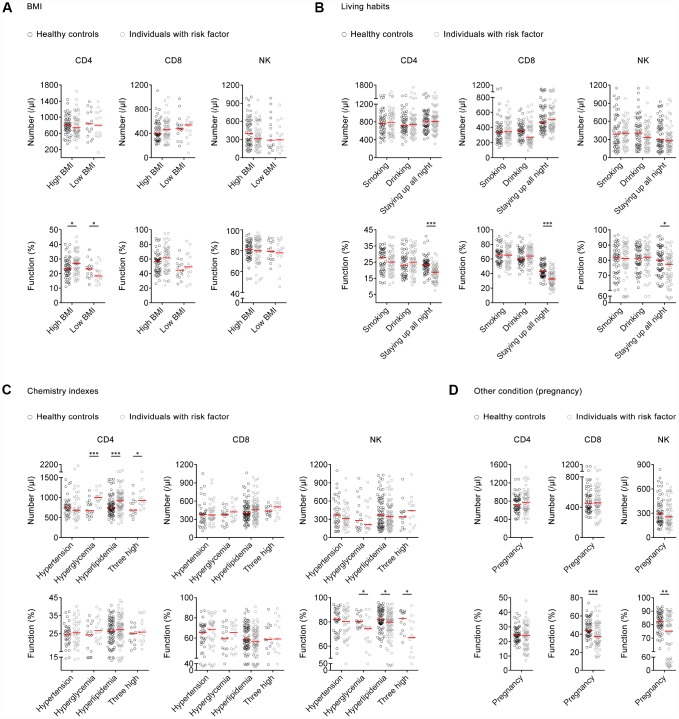
**The effect of risk factors on the number and function of lymphocytes.** (**A**) Scatter plots showing the number and function of CD4^+^, CD8^+^ T cells, and NK cells in high BMI individuals (n=53), low BMI individuals (n=15), and matched healthy controls. (**B**) Scatter plots showing the number and function of CD4^+^, CD8^+^ T cells, and NK cells in individuals with different living habits (smoking, n=50; drinking, n=51; staying up all night, n=59) and matched healthy controls. (**C**) Scatter plots showing the number and function of CD4^+^, CD8^+^ T cells, and NK cells in individuals with different chemistry indexes (hypertension, n=35; hyperglycemia, n=16; hyperlipidemia, n=74; three high, n=13) and matched healthy controls. Three high means hypertension, hyperglycemia, and hyperlipidemia. (**D**) Scatter plots showing the number and function of CD4^+^, CD8^+^ T cells, and NK cells in pregnant women (n=56) and matched healthy controls. Horizontal lines indicate the median. *P < 0.05, **P < 0.01, ***P < 0.001 (Mann–Whitney *U*-test). BMI, body mass index.

A total of 422 (male, 46.45%) participants with different risk factors were recruited. Demographic characteristics of individuals with different risk factors and matched healthy controls are shown in Supplementary Table 4. For body mass index (BMI), there was no significant difference in the number of lymphocytes in individuals with high or low BMI compared with matched healthy controls. Differently, the function of CD4^+^ T cells was significantly increased or decreased in individuals with high or low BMI respectively, compared with matched healthy controls. There was no significant difference in the function of CD8^+^ T cells and NK cells either between high BMI individuals and healthy controls or between low BMI individuals and healthy controls (Figure 4A).

For living habits, there was no significant difference in the number and function of lymphocytes between individuals who drank or smoked and those who did not. However, the function of CD4^+^, CD8^+^ T cells, and NK cells was significantly decreased in individuals who stayed up all night compared with those who did not (Figure 4B).

For chemistry indexes, there was significantly higher number of CD4^+^ T cells in individuals with hyperglycemia, hyperlipidemia, or combined conditions, compared with matched healthy controls. No significant differences were found in the number of CD8^+^ T cells and NK cells between individuals with hyperglycemia, hyperlipidemia, hypertension, or combined conditions and matched healthy controls. Meanwhile, no significant difference was found in the function of CD4^+^ and CD8^+^ T cells between individuals with these risk factors and healthy controls. A significantly decreased function of NK cells was found in individuals with hyperglycemia, hyperlipidemia, or combined conditions compared with matched healthy controls, while NK cell function had no difference between individuals with hypertension and controls (Figure 4C).

Regarding pregnancy, there was no significant difference in the number of lymphocytes between pregnant women and matched non-pregnant controls. However, the function of CD8^+^ T cells and NK cells was significantly decreased in pregnant women compared with non-pregnant controls (Figure 4D).

### The number and function of lymphocytes in different disease models

A total of 683 (male, 54.32%) participants with various disease models including immunosuppressive conditions, autoimmune diseases, and opportunistic infections were recruited. Demographic characteristics of patients and matched healthy controls are shown in Supplementary Table 4.

**Figure 5:**
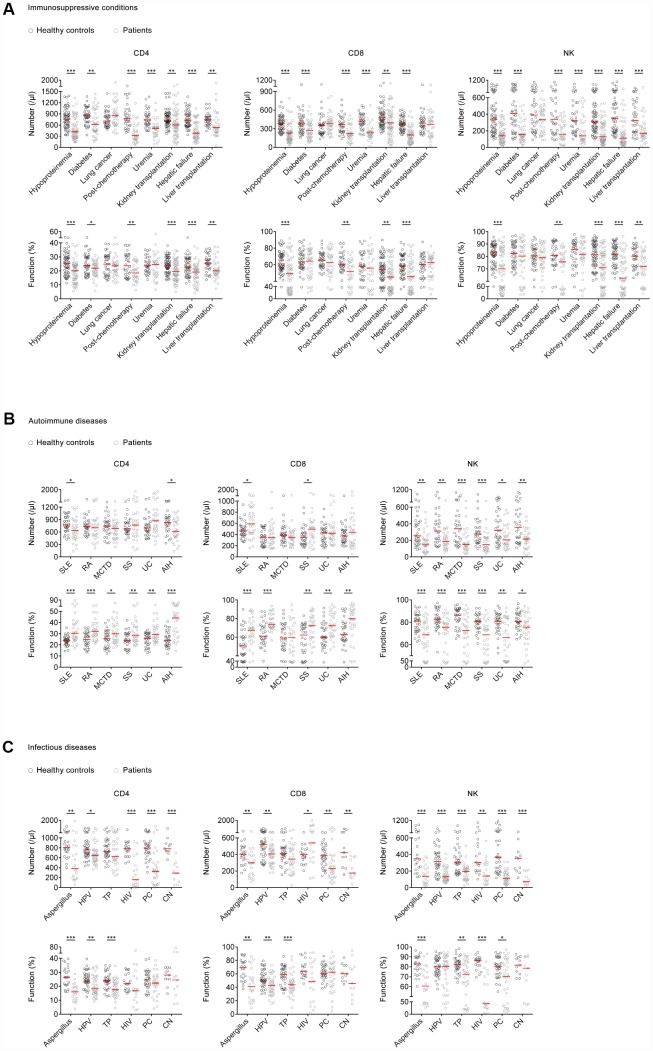
**The number and function of lymphocytes in different disease models.** (**A**) Scatter plots showing the number and function of CD4^+^, CD8^+^ T cells, and NK cells in patients with hypoproteinemia (n=58), diabetes (n=40), lung cancer (n=37), post-chemotherapy (n=32), uremia (n=33), kidney transplantation (n=61), hepatic failure (n=49), liver transplantation (n=33), and matched healthy controls. (**B**) Scatter plots showing the number and function of CD4^+^, CD8^+^ T cells, and NK cells in patients with SLE (n=33), RA (n=30), MCTD (n=31), UC (n=31), SS (n=30), AIH (n=30), and matched healthy controls. (**C**) Scatter plots showing the number and function of CD4^+^, CD8^+^ T cells, and NK cells in patients with aspergillus infection (n=22), HPV infection (n=38), TP infection (n=34), HIV infection (n=19), PC infection (n=31), CN infection (n=11), and matched healthy controls. Horizontal lines indicate the median. *P < 0.05, **P < 0.01, ***P < 0.001 (Mann–Whitney *U*-test). SLE, systemic lupus erythematosus; RA, rheumatoid arthritis; MCTD, mixed connective tissue diseases; UC, ulcerative colitis; SS, Sjögren syndrome; AIH, autoimmune hepatitis; HPV, human papillomavirus; TP, *Treponema pallidum*; HIV, human immunodeficiency virus; PC, *Pneumocystis carinii*; CN, *Cryptococcus neoformans*.

Patients with immunosuppressive conditions including hypoproteinemia, post-chemotherapy, kidney transplantation, and hepatic failure had both significantly lower number and lower function of CD4^+^, CD8^+^ T cells, and NK cells than healthy controls. No significant difference was found in the function of CD4^+^, CD8^+^ T cells, and NK cells between patients with lung cancer or uremia and healthy controls (Figure 5A).

Patients with autoimmune diseases including systemic lupus erythematosus (SLE) or autoimmune hepatitis (AIH) showed significantly lower number of CD4^+^ T cells than healthy controls, while the number of CD4^+^ T cells in patients with rheumatoid arthritis (RA), mixed connective tissue disease (MCTD), Sjögren syndrome (SS), or ulcerative colitis (UC) was normal. Patients with SLE or SS showed significantly higher number of CD8^+^ T cells than healthy controls, while the number of CD8^+^ T cells in patients with RA, MCTD, UC, or AIH was normal. On the other hand, the function of CD4^+^ and CD8^+^ T cells in patients with most autoimmune diseases was significantly increased compared with that in healthy controls. Differently, patients with all these autoimmune diseases showed both significantly lower number and lower function of NK cells than healthy controls (Figure 5B).

Patients with opportunistic infections such as human papillomavirus (HPV), HIV, aspergillus, *Pneumocystis carinii* (PC), and *Cryptococcus neoformans* (CN) infection showed significantly lower number of CD4^+^ T cells and NK cells than healthy controls. In contrast, the number of CD8^+^ T cells in patients with HIV infection was significantly increased compared with that in healthy controls. On the other hand, patients with infectious diseases such as HPV, *Treponema pallidum* (TP), and aspergillus infection showed significantly lower function of CD4^+^ and CD8^+^ T cells than healthy controls. Although a decreased trend was found in CD4^+^ and CD8^+^ T cell function in patients with HIV, PC, and CN infection, no significant difference was observed between these patients and healthy controls (Figure 5C).

### Monitoring of the number and function of lymphocytes in kidney transplant recipients

A total of 35 (male, 65.71%) patients who underwent kidney transplantation were recruited and followed up for 6 months after transplantation. Demographic and clinical characteristics of these transplant recipients are shown in Supplementary Table 5.

**Figure 6:**
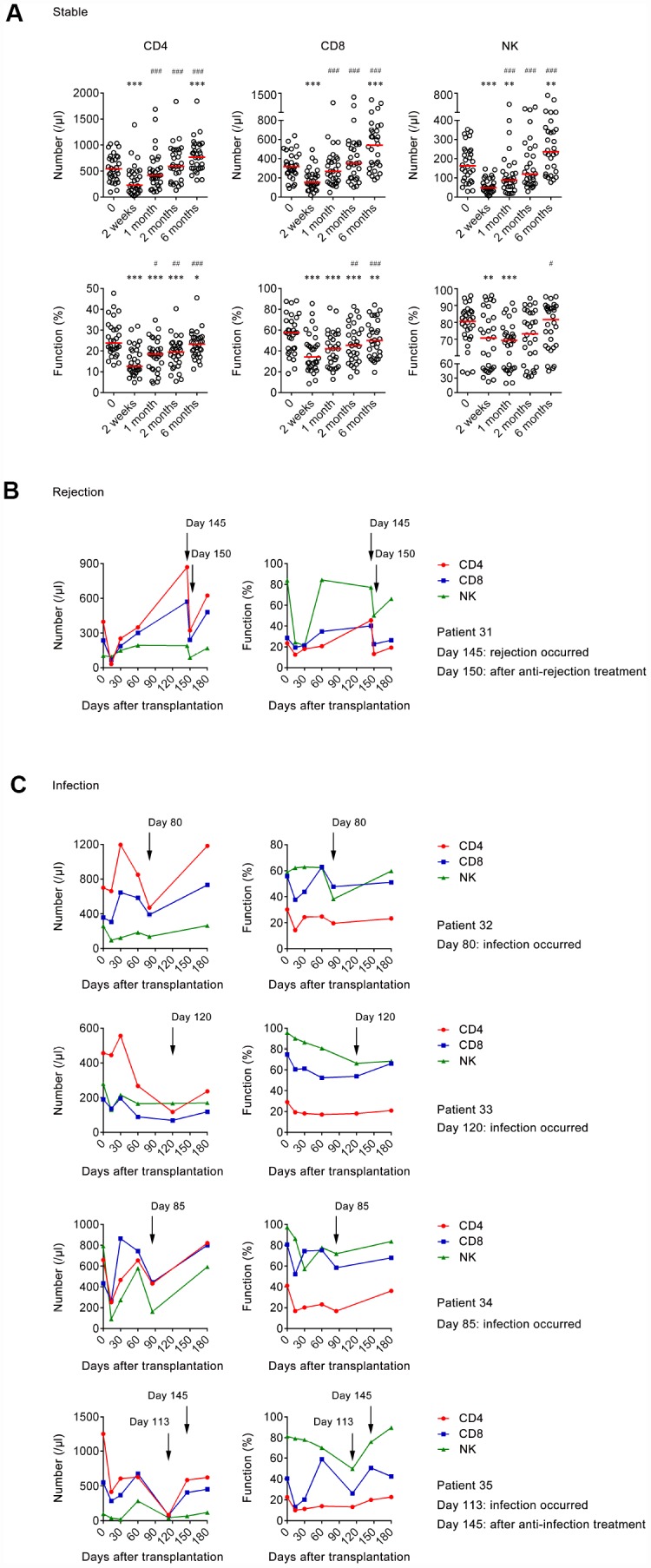
**The number and function of lymphocytes in transplant recipients at different time points.** (**A**) Scatter plots showing the number and function of CD4^+^, CD8^+^ T cells, and NK cells in stable group (n=30) at pretransplantation, 2 weeks, 1 month, 2 months, and 6 months after transplantation. Horizontal lines indicate the median. *P < 0.05, **P < 0.01, ***P < 0.001 (Wilcoxon test) (compared with pretransplantation). ^#^*P* < 0.05, ^##^*P* < 0.01, ^###^*P* < 0.001 (Wilcoxon test) (compared with 2 weeks after transplantation). (**B**) Line diagrams showing the number and function of CD4^+^, CD8^+^ T cells, and NK cells at different days after transplantation in 1 rejection patient. (**C**) Line diagrams showing the number and function of CD4^+^, CD8^+^ T cells, and NK cells at different days after transplantation in 4 infection patients.

Of 35 transplant recipients, 30 were in a stable state. In these patients, the number of CD4^+^, CD8^+^ T cells, and NK cells was significantly decreased after 2 weeks of transplantation compared with that of pretransplantation. Then, the number of CD4^+^, CD8^+^ T cells, and NK cells was gradually increased. At 6 months post-transplantation, the number of these cells was significantly higher than that of pretransplantation. Similarly, the function of CD4^+^, CD8^+^ T cells, and NK cells was also significantly decreased after 2 weeks of transplantation compared with that of pretransplantation and then gradually restored (Figure 6A). The results of the number and function of 30 recipients with stable state are shown in Supplementary Figure 3.

Acute rejection occurred in one transplant recipient (patient 31) approximately at 5 months post-transplantation. The number of CD4^+^ and CD8^+^ T cells was increased at the time of rejection compared with at 2 months post-transplantation. The function of CD4^+^ T cells also had an increased trend when rejection occurred, while the function of CD8^+^ T cells and NK cells seemed to have no change. After 5 days of anti-rejection treatment, the number and function of CD4^+^, CD8^+^ T cells, and NK cells were all obviously decreased (Figure 6B).

Infection occurred in 4 transplant recipients (patient 32-35) during 2-5 months post-transplantation. The number and function of CD4^+^, CD8^+^ T cells, or NK cells in all four patients were decreased in a different degree when infection occurred. Furthermore, both the number and function of CD4^+^, CD8^+^ T cells, and NK cells in patient 35 returned to pre-infection level after one month of anti-infection treatment (Figure 6C).

## DISCUSSION

Evaluation of host lymphocyte function has always been the focus of attention [1, 4, 17, 18]. Currently, clinicians usually assess patients’ lymphocyte function based on whether patients have underlying diseases, and this method has a lot of inaccuracy. Although several studies have shown that traditional methods such as [^3^H]-thymidine incorporation, (^51^Cr)-release test, and carboxyfluorescein diacetate succinimidyl ester (CFSE) labeling assay may have potential value in this field [19–23]. However, there is no method available to quantitatively detect the function of lymphocytes in clinical practice.

We have previously established a method based on analysis of the IFN-γ producing capability of lymphocytes under PMA/Ionomycin stimulation, which can be used to detect the function of CD4^+^, CD8^+^ T cells, and NK cells simultaneously [16]. In this study, we further optimized the protocol of this method. The reproducibility of this assay was examined and we found the CVs of the assay to be satisfactory. This assay is simple and reproducible, and is of great value in clinical practice.

The relationship between lymphocyte number and age has been widely reported [24–27], while there are few studies that determine the relationship between lymphocyte function and age [28–30]. Furthermore, there are rare studies that explore the relationship between lymphocyte number and function. In accordance with previous studies, we found the number of CD4^+^ and CD8^+^ T cells was gradually decreased with increasing age but the number of NK cells had an increased trend in old people [25, 31, 32]. Interestingly, we found there was almost no function of CD4^+^ and CD8^+^ T cells in infants just after birth. The function of CD4^+^ T cells was gradually increased with increasing age and maintained at a certain level in adults. The function of CD8^+^ T cells even increased during the entire lifetime, which may be caused by the continuous decrease of cell number. Surprisingly, the function of NK cells was maintained at a high level after birth, which suggests that innate immunity is developed well in embryonic period. Based on these data, we speculate that the impairment of immunity in children is caused by dysfunction of CD4^+^ and CD8^+^ T cells, while the impairment of that in old people is caused by insufficient number of them. Furthermore, the reason why the number of NK cells is increased in old people may be for making compensation for decreased adaptive immunity. These data confirmed that the development of innate and adaptive immunity is different during lifetime. In addition to the above, one of the most important innovations in this study was that we revealed the correlation between the number and function of lymphocytes. In general, a significantly negative correlation existed between the number and function of both CD4^+^ and CD8^+^ T cells, which may indicate that the number and function of lymphocytes are both critical to maintain normal host immunity.

Furthermore, we accidentally found that lymphocytes with different expression levels of CD4 or CD8 showed different IFN-γ-producing ability upon PMA/Ionomycin stimulation. Specifically, high CD4 expression T cell subset had higher function than low CD4 expression T cell subset. Conversely, high CD8 expression T cell subset was weaker than low CD8 expression T cell subset. No difference was found in the function of NK cells between different CD56 expression subsets (Supplementary Figure 4). These data suggest that the development of CD4^+^ T cells and CD8^+^ T cells may be also different. However, we did not investigate the mechanism behind this phenomenon and further exploration is needed.

Regarding the effect of risk factors on host immunity, we reveal that moderate obesity may enhance host immunity while weight loss will weaken it, which is in accordance with traditional views that host immunity is impaired in malnutrition people [33–35]. The abnormal metabolism of sugar and lipid seems to have no effect on T cell function but inhibits NK cell function. Surprisingly, staying up all night impairs the function of CD4^+^, CD8^+^ T cells, and NK cells simultaneously, which confirms that adequate sleep is critical factor for host immunity. The pregnant women show impaired function of CD8^+^ T cells and NK cells, which is in accordance with previous studies showing that pregnant women have a higher risk of developing infectious diseases [36–38]. These data suggest that some risk factors may not affect lymphocyte number but significantly impair their function, which results in decreased host immunity.

The traditional view is that patients with underlying diseases have impaired immunity [39–43]. However, there is rare experimental evidence supporting this. Our data showed that patients with some underlying diseases such as organ transplantation and hypoproteinemia had decreased lymphocyte number and function. Although the function of lymphocytes in uremic patients is normal, the number of them in these patients is actually at a very low level. So, the overall immunity of uremic patients is still low. These data suggest that many underlying diseases can damage lymphocyte immunity, either in cell number or in cell function. On the contrary, the number of CD4^+^ and CD8^+^ T cells in many autoimmune diseases has no change, but actually the function of them is very high, which eventually leads to a hyper-state of adaptive immunity. As we know, patients infected with opportunistic pathogens shows impaired host immunity. Surprisingly, we found that some patients infected with HIV or PC had a high CD4^+^ T cell function, but actually the number of CD4^+^ T cells in these patients was very low. Thus, the overall host immunity in these patients should be still considered as low. These data suggest that combination of lymphocyte number and function play an important role in evaluating the overall immunity of patients with immune-related diseases.

The strengths of our study include the following: (1) the age distribution of included participants is comprehensive (from birth to 89 years old); (2) patients with either hyper- or hypo- immune status were recruited; and (3) the inclusion criteria of participants are strict, and using matched healthy controls ensures the credibility of the results. Some limitations of this study should be noted. First, the number of individuals in a single group is relatively small. Second, since all included subjects are Han Chinese in central China, the intervals of lymphocyte number and function established in this article may not be applicable to other areas.

Overall, our study confirms the excellent clinical utility of the combination of lymphocyte number and function in evaluating host immunity, which provide new strategies for the diagnosis, treatment, and prognosis of immune-related diseases.

## MATERIALS AND METHODS

### Participants

This study was carried out in accordance with the recommendations of ethical committee of Tongji Hospital, Tongji Medical College, Huazhong University of Science and Technology. All subjects gave written informed consent with the Declaration of Helsinki.

The healthy individuals (from 0 to 89 years old) who did not show any clinical symptoms were recruited from Tongji Hospital between January 2018 and April 2019. Exclusion criteria of healthy individuals were as follows: smoking, drinking, staying up all night, abnormal BMI, pregnancy, atherosclerosis and vascular disease, cardiopathy, chronic nephropathy, hepatobiliary disease, allergic disease, autoimmune disease, hematological disease, myopathy, burns and muscle trauma, positive for HIV, HBV, HCV, and syphilis antibodies, and receiving medical treatment.

Individuals with different risk factors including abnormality of BMI, abnormality of chemistry indexes, different living habits, and pregnancy were recruited. Patients with immunosuppressive conditions, autoimmune diseases, and opportunistic infections were also recruited. Individuals with immunosuppressive conditions were defined as patients with diabetes, hepatic failure, or patients with solid organ transplantation and receiving immunosuppressant treatment. Patients with autoimmune diseases were defined according to the criteria of the American College of Rheumatology or other international standards [44–53]. Patients with opportunistic infections were defined as individuals infected with pathogens such as aspergillus, PC, and CN. The definitions of the above-mentioned states are shown in Supplementary Table 6**.**

Participants in healthy control, risk factor, and disease groups were matched for the following criteria: gender-consistency, age (± 3 years), and 1:1 pairing. Entry criteria of these healthy controls were consistent with the criteria of healthy participants described as above.

The kidney transplant recipients were recruited and underwent continuous monitoring of the number and function of lymphocytes. The time points of the tests were before surgery, 2 weeks, 1 month, 2 months, and 6 months after surgery. When patients were suspected as having rejection or infection, lymphocyte number and function assay were performed immediately. The transplant recipients were classified as stable state, rejection, and infection after transplantation. Recipients with rejection were confirmed by pathological examination. Recipients with infection were confirmed by microbial findings, or by subsequent treatment response in those who had no evidence of pathogens.

### PMA/Ionomycin-stimulated lymphocyte function assay

PMA/ionomycin-stimulated lymphocyte function assay was performed as described previously [16]. The procedures are described in brief as following: (1) 100 μl of whole blood was diluted with 400 μl of IMDM medium; (2) the diluted whole blood was incubated in the presence of Leukocyte Activation Cocktail (Becton Dickinson GolgiPlug™) for 4 h; (3) the cells were labeled with antibodies (anti-CD45, anti-CD3, anti-CD4, anti-CD56, and anti-CD8); (4) the cell were fixed and permeabilized; (5) the cells were stained with intracellular anti-IFN-γ antibody; and (6) the cells were analyzed with FACSCanto flow cytometer. The percentages of IFN-γ^+^ cells in different cell subsets were defined as the function of them (e.g., the percentage of IFN-γ^+^ cells in CD3^+^CD4^+^CD8^-^ cells was regarded as the function of CD4^+^ T cells; the percentage of IFN-γ^+^ cells in CD3^+^CD4^-^CD8^+^ cells was regarded as the function of CD8^+^ T cells; the percentage of IFN-γ^+^ cells in CD3^-^CD56^+^ cells was regarded as the function of NK cells).

### Lymphocyte subset count

The total number of lymphocytes in peripheral blood was counted by hemocytometer. The percentages of CD3^+^CD4^+^CD8^-^, CD3^+^CD4^-^CD8^+^, and CD3^-^CD56^+^ cells among the total lymphocytes were obtained as above lymphocyte function assay. The absolute numbers of different lymphocyte subsets were calculated by multiplying the percentages with total lymphocyte count (CD4^+^ T cell count = total lymphocyte count × CD3^+^CD4^+^CD8^-^%, CD8^+^ T cell count = total lymphocyte count × CD3^+^CD4^-^CD8^+^%, NK cell count = total lymphocyte count × CD3^-^CD56^+^%).

### Statistics

Data were analyzed using GraphPad Prism 6.0 (GraphPad, La Jolla, CA, USA). Differences between groups were analyzed using the Mann-Whitney *U*-test or Wilcoxon test. Statistical significance was determined as *P* < 0.05.

## Supplementary Material

Supplementary Figures

Supplementary Tables

Supplementary Table 3
