# Supplementary material for: Combination of lymphocyte number and function in evaluating host immunity
Source: Aging (Albany NY). 2019 Dec 20;11(24):12685–707. doi: 10.18632/aging.102595 (PMC6949078; doi:10.18632/aging.102595)
Supplement: Supplementary Figures [file aging-11-102595-s003..pdf]

SUPPLEMENTARY FIGURES

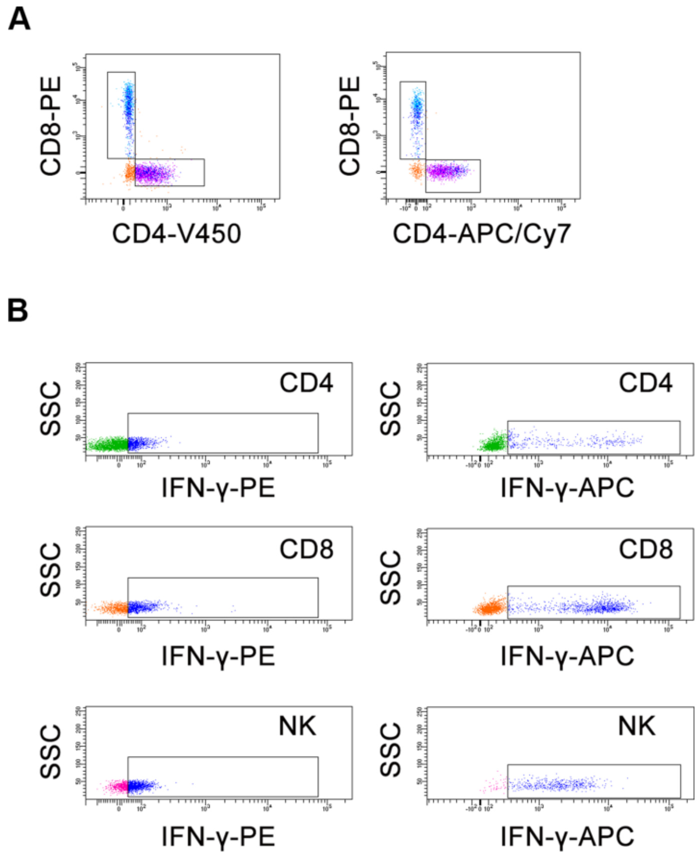

Supplementary Figure 1. The optimization of antibody combination of lymphocyte function assay. Diluted whole blood was stimulated with Leukocyte Activation Cocktail for 4 h. (**A**) Representative flow plots showing the results of the same sample labeled with anti-CD4-V450 and anti-CD4-APC-Cy7. (**B**) Representative flow plots showing the results of the same sample labeled with anti-IFN-γ-PE and anti-IFN-γ-APC. APC, allophycocyanin; PE, phycoerythrin.

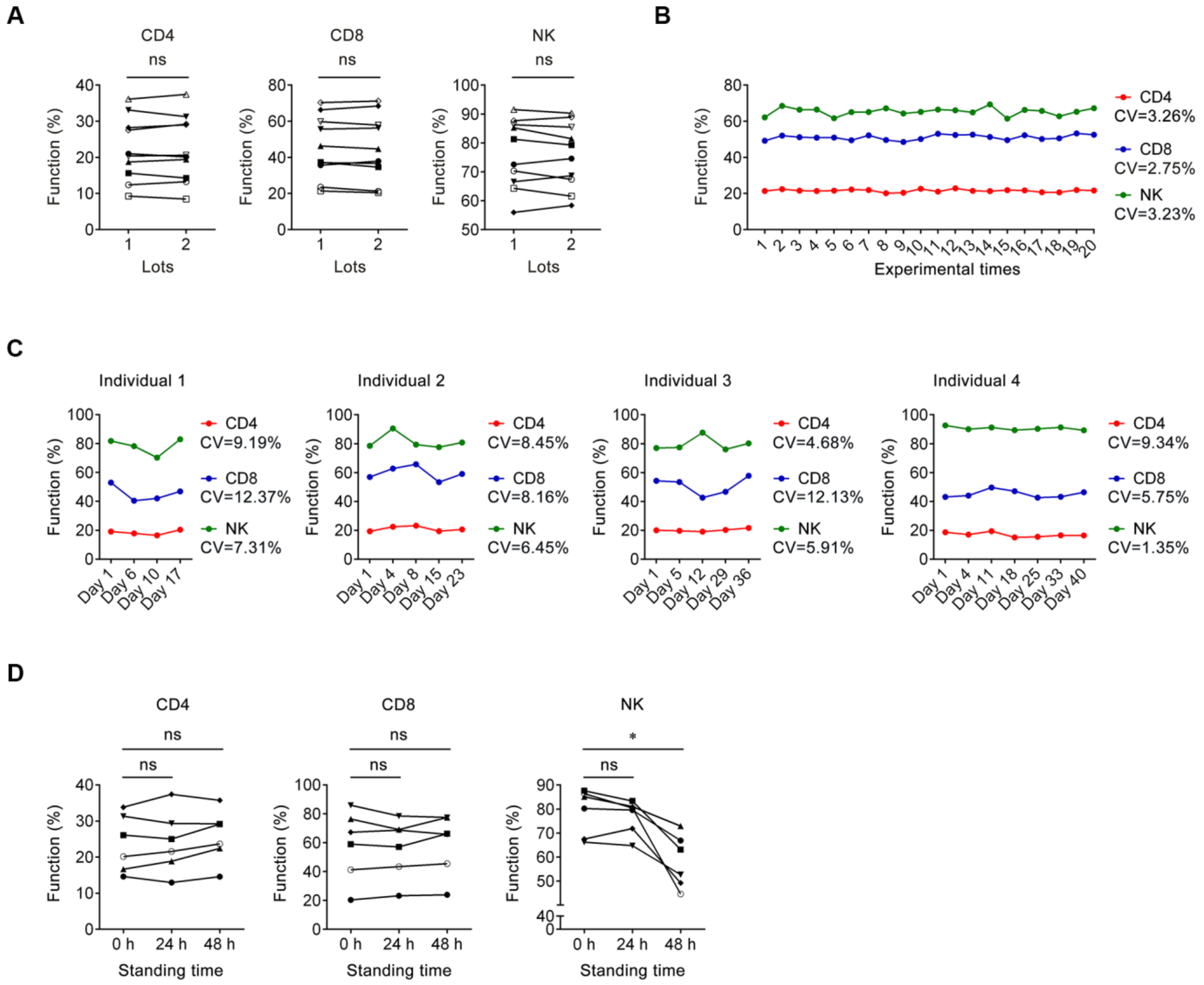

Supplementary Figure 2. Performance verification of lymphocyte function assay. (**A**) Lymphocyte function assay was performed in 10 individuals by using two batches of reagents. Line diagrams showing the function of $CD4^+$, $CD8^+$ T cells, and NK cells in these individuals. Each line represents an individual donor. ns, no significance (Wilcoxon test). (**B**) The intra-assay repeatability of lymphocyte function assay was evaluated by analyzing 20 replicates. Line diagrams showing the function of $CD4^+$, $CD8^+$ T cells, and NK cells. (**C**) Lymphocyte function assay was performed in 4 individuals at different days. Line diagrams showing the function of $CD4^+$, $CD8^+$ T cells, and NK cells. (**D**) Lymphocyte function assay was performed at 0, 24, or 48 h after specimen collection. Line diagrams showing the function of $CD4^+$, $CD8^+$ T cells, and NK cells. Each line represents an individual donor. *$P < 0.05$, ns, no significance (Wilcoxon test).

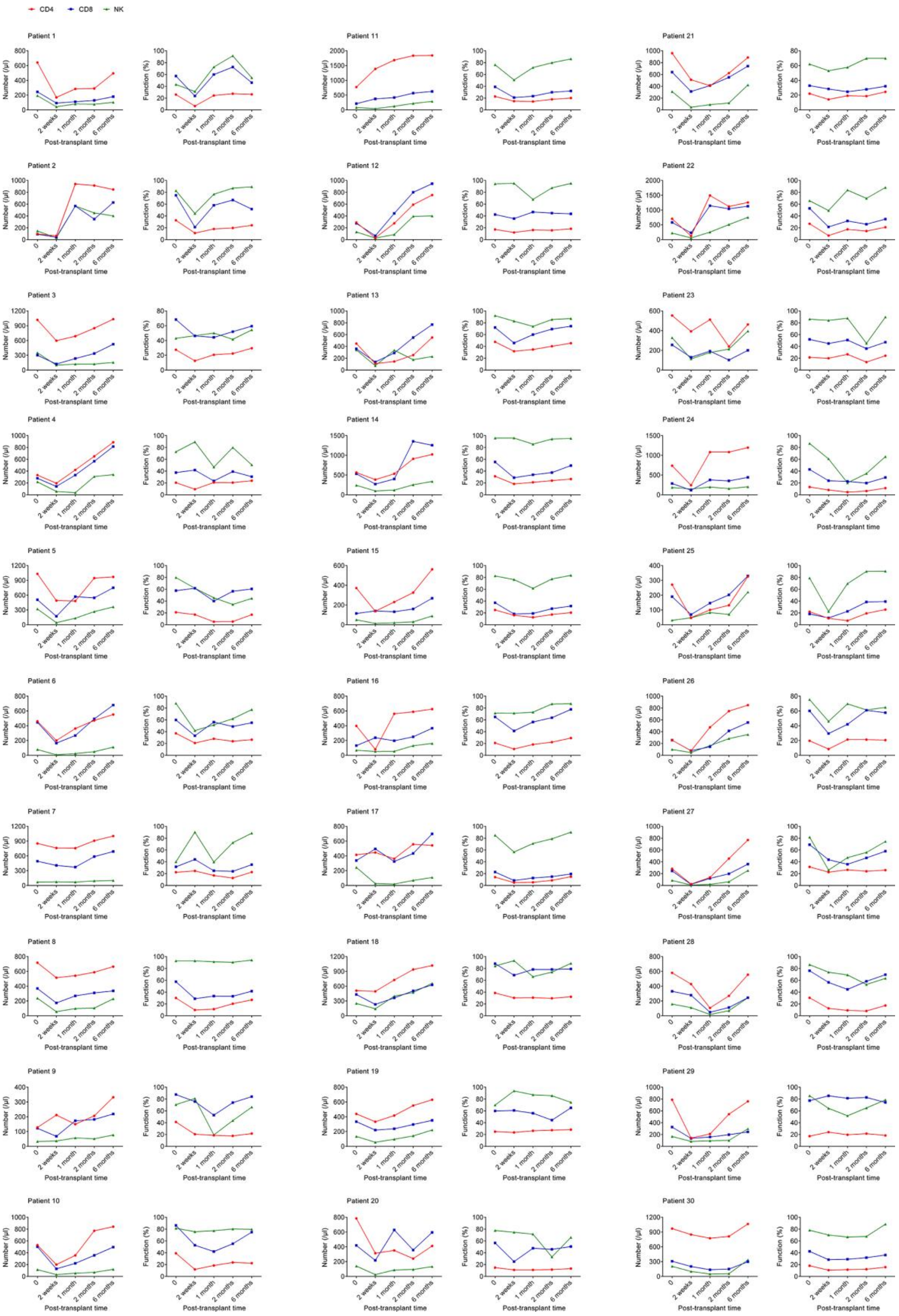

Supplementary Figure 3. The number and function of lymphocytes in stable transplant recipients. Line diagrams showing the number and function of $CD4^+$, $CD8^+$ T cells, and NK cells in stable transplant recipients (n=30) at pretransplantation, 2 weeks, 1 month, 2 months, and 6 months after transplantation.

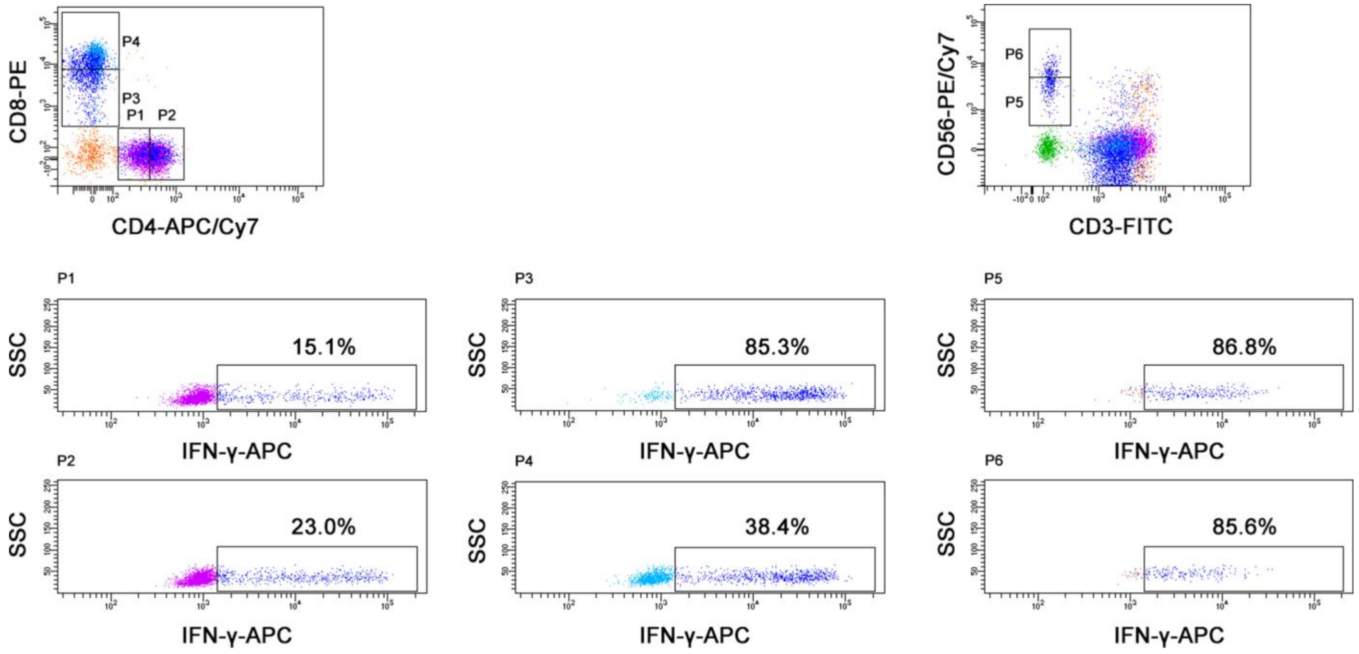

Supplementary Figure 4. Lymphocyte function in cells with different CD4, CD8 and CD56 expression levels. Diluted whole blood was stimulated with Leukocyte Activation Cocktail for 4 h. Representative flow cytometry gating strategies for identification of IFN-γ^+ cells in lymphocytes with low or high CD4, CD8, and CD56 expression levels.
