# Supplementary material for: Combination of lymphocyte number and function in evaluating host immunity
Source: Aging (Albany NY). 2019 Dec 20;11(24):12685–707. doi: 10.18632/aging.102595 (PMC6949078; doi:10.18632/aging.102595)
Supplement: Supplementary Tables [file aging-11-102595-s002..pdf]

SUPPLEMENTARY TABLES

Supplementary Table 1. Characteristics of antibodies used in flow cytometry.

Marker	Fluorochrome	Manufacturer	Catalog number
CD45	PerCp	Becton Dickinson	652803
CD3	FITC	Becton Dickinson	349201
CD4	APC-Cy7	Becton Dickinson	341115
CD8	PE	Becton Dickinson	340046
CD56	PE-Cy7	Becton Dickinson	335791
IFN-γ	APC	Becton Dickinson	554702

Supplementary Table 2. The effect of different lot reagents on lymphocyte function assay.

	Lot 1	Lot 2
CD45	7131048	8283383
CD3	8181542	8335832
CD4	8227974	8335846
CD8	8080642	8226704
CD56	8142716	8347692
IFN-γ	7121607	8086695
Stimulant	7265717	8088666

Please browse Full Text version to see the data of Supplementary Table 3.

Supplementary Table 3. The intervals of lymphocyte number and function in healthy individuals between 0 and 89 years of age.

Supplementary Table 4. Demographic characteristics of individuals with different states and healthy controls.

States			Experimental number (male %)	Ages (years, means ± SD)		*p**
				Experimental	Control	
Risk factors	BMI	High BMI	53 (67.9%)	44.3 ± 16.2	44.7 ± 16.0	0.886
		Low BMI	15 (53.3%)	35.3 ± 17.0	36.0 ± 16.6	0.644
	Chemistry indexes	Hyperglycemia	16 (50.0%)	52.2 ± 11.3	52.2 ± 11.3	1
		Hyperlipidemia	74 (59.5%)	47.5 ± 10.0	47.5 ± 10.0	0.992
		Hypertension	35 (54.3%)	53.8 ± 15.7	54.0 ± 15.7	0.942
		Three high	13 (84.6%)	51.6 ± 10.1	50.9 ± 10.1	0.771
	Living habits	Smoking	50 (76.0%)	56.1 ± 8.6	55.9 ± 9.2	0.87
		Drinking	51 (62.7%)	56.3 ± 7.4	56.2 ± 7.4	0.882
		Staying up all night	59 (0.0%)	25.0 ± 2.0	25.4 ± 1.9	0.177
	Pregnancy	Pregnancy	56 (0.0%)	29.2 ± 4.3	29.4 ± 4.4	0.67
Diseases	Immunosuppressive conditions	Hypoproteinemia	58 (72.4%)	52.9 ± 11.9	52.8 ± 12.0	0.922
		Uremia	33 (66.7%)	46.1 ± 15.0	46.9 ± 15.8	0.667
		Hepatic failure	49 (77.6%)	49.9 ± 12.6	49.6 ± 12.6	0.903
		Kidney transplantation	61 (65.6%)	40.9 ± 10.9	40.0 ± 10.9	0.502
		Liver transplantation	33 (69.7%)	48.5 ± 9.6	48.1 ± 10.0	0.851
		Lung cancer	37 (40.5%)	58.6 ± 7.8	58.7 ± 8.0	0.946
		Post-chemotherapy	32 (53.1%)	55.0 ± 9.7	55.2 ± 10.4	0.992
		Diabetes	40 (60.0%)	58.2 ± 12.2	58.0 ± 12.0	0.816
	Autoimmune diseases	Systemic lupus erythematosus	33 (18.2%)	38.6 ± 14.0	37.5 ± 13.0	0.733
		Rheumatoid arthritis	30 (30.0%)	55.2 ± 11.1	54.9 ± 11.8	0.851
		Ulcerative colitis	31 (58.1%)	49.2 ± 12.1	49.3 ± 12.1	0.941
		Autoimmune hepatitis	30 (33.3%)	57.5 ± 11.1	57.2 ± 11.3	0.991
		Sjögren syndrome	30 (13.3%)	45.1 ± 15.6	44.7 ± 15.7	0.95
		Mixed connective tissue disease	31 (48.4%)	51.5 ± 12.0	51.5 ± 12.1	1
	Infection	Aspergillus infection	22 (59.1%)	52.0 ± 14.7	51.8 ± 14.9	0.986
		HPV infection	38 (63.2%)	35.7 ± 9.6	35.2 ± 10.1	0.802
		TP infection	34 (26.5%)	45.0 ± 17.0	45.2 ± 17.6	0.993
		HIV infection	19 (84.2%)	52.0 ± 14.0	52.0 ± 14.0	0.971
		PC infection	31 (74.2%)	51.6 ± 14.2	51.6 ± 14.7	0.992
		CN infection	11 (81.8%)	46.7 ± 16.2	46.9 ± 16.3	0.858

BMI, body mass index; HPV, human papillomavirus; TP, Treponema pallidum; HIV, human immunodeficiency virus; PC, Pneumocystis carinii; CN, Cryptococcus neoformans. Three high means hypertension, hyperglycemia, and hyperlipidemia. *Comparisons were performed between the experimental and control groups using Mann–Whitney *U* test. Data are presented as means ± SD, or number (male %).

Supplementary Table 5. Demographic and clinical characteristics of kidney transplant recipients.

Patient number	Gender	Ages (years)	Post-transplant status	Complement
1	M	44	Stable	NA
2	M	50	Stable	NA
3	M	52	Stable	NA
4	M	34	Stable	NA
5	M	37	Stable	NA
6	F	43	Stable	NA
7	M	24	Stable	NA
8	F	51	Stable	NA
9	M	48	Stable	NA
10	M	61	Stable	NA
11	F	32	Stable	NA
12	F	23	Stable	NA
13	M	48	Stable	NA
14	M	53	Stable	NA
15	M	29	Stable	NA
16	M	55	Stable	NA
17	M	24	Stable	NA
18	M	31	Stable	NA
19	M	33	Stable	NA
20	M	30	Stable	NA
21	M	30	Stable	NA
22	F	31	Stable	NA
23	M	59	Stable	NA
24	F	42	Stable	NA
25	F	16	Stable	NA
26	F	46	Stable	NA
27	F	50	Stable	NA
28	F	66	Stable	NA
29	M	51	Stable	NA
30	M	55	Stable	NA
31	M	64	Rejection	Acute rejection was confirmed by pathological examination.
32	M	49	Infection	The patient had a cough and chest computed tomography scan indicated the presence of infection. The patient was relieved of his symptoms after antibiotic treatment (moxifloxacin and ceftriaxone).
33	F	52	Infection	The patient had a cough and chest computed tomography scan indicated the presence of infection. The patient was relieved of her symptoms after antibiotic treatment (cefoperazone/tazobactam).
34	M	44	Infection	The patient had fever (38.5℃) and CMV DNA was positive in blood. Chest computed tomography scan indicated the presence of infection and interstitial changes. The patient was relieved of his symptoms after antibiotic treatment and antiviral treatment (cefoperazone/tazobactam and ganciclovir).
35	F	29	Infection	The patient had a cough and chest computed tomography showed lung shadow. *Klebsiella pneumoniae* was isolated from the patient' broncho-alveolar lavage fluid and the patient was relieved of her symptoms after antibiotic treatment (moxifloxacin and cefoperazone/tazobactam).

M, male; F, female; NA, not applicable; CMV, cytomegalovirus.

Supplementary Table 6. Definition for different states.

Classification			Definition
Risk factors	BMI	High BMI	BMI > 23.9
		Low BMI	BMI < 18.5
	Chemistry indexes	Hyperglycemia	Fasting glucose > 6.05 mmol/L
		Hyperlipidemia	TC > 5.18 mmol/L and/or TG > 1.7 mmol/L
		Hypertension	Systolic blood pressure ≥140 mmHg and/or diastolic blood pressure ≥90 mmHg
		Three high	Hypertension, hyperglycemia, and hyperlipidemia exist together
	Living habits	Smoking	Smoking of 5 cigarettes per day on average for more than 10 years
		Drinking	Drinking of 200 ml per day on average for more than 10 years
		Staying up all night	Sleep less than 4 hours in 2 days per week on average in the last 3 months
	Pregnancy	Pregnancy	NA
Diseases	Infection	TP infection	Primary screening test, RPR, and TPPA positive.
		HPV infection	HPV-6 and/or HPV-11 positive.
		HIV infection	HIV antibody positive and confirmed by immunoblotting.
		Aspergillus infection	Positive aspergillus culture of blood.
		CN infection	India-ink capsule staining positive on cerebrospinal fluid, and CN culture positive.
		PC infection	PC DNA positive in broncho-alveolar lavage fluid.
	Immunosuppressive conditions	Uremia	Serum creatinine ≥ 707 μmol/L and creatinine clearance < 10ml/min.
		Hepatic failure	Serum total bilirubin ≥ 171 μmol/L and INR > 1.5.
		Kidney transplantation	Within 6 months after kidney transplantation with regular use of anti-rejection drugs. No infection or rejection occurs.
		Liver transplantation	Within 6 months after liver transplantation with regular use of anti-rejection drugs. No infection or rejection occurs.
		Lung cancer	Roentgenographically occult but endoscopically visible cancer with a surface area < 2 cm and no invasion beyond the bronchial cartilage.
		Post-chemotherapy	Lung cancer patients who have undergone chemotherapy for 3 months.
		Hypoproteinemia	Liver-occupying lesions and serum albumin < 30 g/L.
		Diabetes	Fasting blood glucose ≥ 7 mmol/L and accompanied by other symptoms including diabetic retinopathy, diabetic foot, and/or diabetic nephropathy.
	Autoimmune diseases	Systemic lupus erythematosus	Reference to [48, 53]
		Rheumatoid arthritis	Reference to [44, 45, 46]
		Ulcerative colitis	Reference to [49]
		Autoimmune hepatitis	Reference to [47]
		Sjögren syndrome	Reference to [51, 52]
		Mixed connective tissue disease	Reference to [50]

BMI, body mass index; TC, total cholesterol; TG, triglyceride; NA, not applicable; TP, *Treponema pallidum*; RPR, rapid plasma reagin; TPPA, *Treponema* pallidum particle agglutination assay; HPV, human papillomavirus; HIV, human immunodeficiency virus; CN, *Cryptococcus neoformans*; PC, *Pneumocystis carinii*; INR, international normalized ratio.
