# Supplementary material for: Combination of lymphocyte number and function in evaluating host immunity
Source: Aging (Albany NY). 2019 Dec 20;11(24):12685–707. doi: 10.18632/aging.102595 (PMC6949078; doi:10.18632/aging.102595)
Supplement: Supplementary Table 3 [file aging-11-102595-s001..pdf]

Supplementary Table 3. The intervals of lymphocyte number and function in healthy individuals between 0 and 89 years of age.

CD4

	age (years)	number: total	number: male	number: female	CD4 number (/µl): total	CD4 number (/µl): male	CD4 number (/µl): female	p*	CD4 function (%): total	CD4 function (%): male	CD4 function (%): female	p*
	0	81	42	39	2862 ± 1146 (1143 - 4900)	2723 ± 1092 (1128 - 4890)	3010 ± 1199 (1192 - 5483)	0.315	2.35 ± 1.71 (0.42 - 6.37)	2.34 ± 1.62 (0.41 - 6.19)	2.36 ± 1.82 (0.42 - 6.73)	0.816
	1	29	13	16	2638 ± 874 (1130 - 4345)	2606 ± 919 (1286 - 4146)	2663 ± 866 (974 - 4544)	0.934	6.13 ± 1.88 (2.48 - 9.45)	6.04 ± 2.06 (1.55 - 9.60)	6.21 ± 1.79 (3.40 - 9.30)	0.9
	2	22	14	8	1946 ± 553 (1238 - 3233)	1972 ± 546 (1314 - 3283)	1902 ± 600 (1234 - 2951)	0.643	6.87 ± 1.75 (3.93 - 11.13)	6.84 ± 1.26 (3.95 - 8.64)	6.91 ± 2.50 (3.93 - 11.57)	0.776
	3	22	13	9	1607 ± 675 (663 - 3147)	1646 ± 780 (654 - 3259)	1552 ± 526 (712 - 2514)	0.872	9.30 ± 2.70 (5.04 - 14.65)	8.93 ± 2.75 (5.01 - 14.22)	9.85 ± 2.69 (6.03 - 14.72)	0.428
	4	22	12	10	1508 ± 586 (545 - 2381)	1488 ± 583 (543 - 2182)	1532 ± 619 (863 - 2414)	0.859	12.47 ± 2.95 (7.10 - 16.90)	13.10 ± 2.21 (9.21 - 15.03)	11.72 ± 3.63 (7.10 - 16.90)	0.198
	5	26	11	15	1275 ± 321 (657 - 1848)	1265 ± 282 (823 - 1638)	1282 ± 356 (567 - 1931)	0.981	12.50 ± 2.59 (9.04 - 17.62)	13.07 ± 3.05 (9.23 - 17.64)	12.07 ± 2.21 (8.94 - 16.68)	0.381
	6	25	14	11	1425 ± 443 (669 - 2127)	1290 ± 387 (649 - 2032)	1598 ± 467 (716 - 2167)	0.075	14.02 ± 2.88 (9.59 - 19.37)	14.05 ± 2.99 (9.32 - 18.35)	13.97 ± 2.87 (10.91 - 19.81)	0.834
	7	21	12	9	1209 ± 461 (593 - 2087)	1181 ± 386 (601 - 2094)	1246 ± 569 (592 - 2024)	0.839	15.35 ± 3.04 (10.27 - 20.93)	15.53 ± 3.20 (10.13 - 21.04)	15.11 ± 3.00 (11.56 - 19.25)	0.734
	8	23	9	14	1145 ± 257 (777 - 1582)	1125 ± 259 (812 - 1503)	1157 ± 265 (768 - 1600)	0.714	14.68 ± 3.02 (10.55 - 21.47)	14.78 ± 3.12 (10.30 - 18.93)	14.62 ± 3.08 (11.57 - 22.11)	0.917
	9	20	10	10	1063 ± 209 (724 - 1455)	1023 ± 202 (808 - 1378)	1103 ± 219 (720 - 1459)	0.404	15.15 ± 2.88 (9.54 - 20.04)	14.78 ± 2.91 (12.17- 19.97)	15.52 ± 2.96 (9.40 - 20.04)	0.382
	10	21	12	9	1192 ± 280 (747 - 1850)	1090 ± 234 (747 - 1437)	1329 ± 289 (1007 - 1864)	0.128	15.56 ± 2.31 (11.51 - 20.17)	15.24 ± 1.86 (11.50 - 17.54)	16.00 ± 2.87 (11.62 - 20.26)	0.851
	11	20	7	13	1033 ± 207 (581 - 1345)	999 ± 244 (572 - 1330)	1051 ± 193 (758 - 1346)	0.42	15.63 ± 4.22 (10.97 - 24.31)	15.98 ± 4.45 (11.00 - 24.31)	15.45 ± 4.27 (10.97 - 24.27)	0.695
0-89	12	23	13	10	1077 ± 283 (703 - 1716)	1032 ± 287 (699 - 1603)	1137 ± 282 (770 - 1744)	0.282	15.03 ± 4.22 (10.35 - 25.86)	15.86 ± 5.01 (10.33- 26.63)	13.95 ± 2.79 (10.83 - 20.92)	0.51
	13	22	12	10	1059 ± 225 (772 - 1565)	1001 ± 245 (770 - 1588)	1128 ± 188 (785 - 1433)	0.122	15.64 ± 5.22 (10.05 - 25.41)	16.94 ± 4.94 (10.50 - 25.20)	14.06 ± 5.36 (10.04 - 25.45)	0.157
	14	22	8	14	1091 ± 256 (590 - 1500)	1067 ± 270 (587 - 1434)	1106 ± 257 (609 - 1508)	0.726	16.98 ± 2.25 (13.38 - 21.64)	16.69 ± 2.64 (13.43 - 21.65)	17.14 ± 2.10 (13.37 - 21.57)	0.507
	15	21	11	10	952 ± 299 (402 - 1495)	851 ± 296 (375 - 1501)	1062 ± 276 (717 - 1444)	0.084	18.95 ± 3.69 (12.61 - 24.93)	19.68 ± 3.65 (12.60 - 25.20)	18.13 ± 3.74 (12.73 - 22.47)	0.309
	16	20	9	11	1006 ± 213 (634 - 1337)	989 ± 198 (711 - 1340)	1021 ± 233 (632 - 1274)	0.692	20.19 ± 4.21 (14.93 - 27.83)	19.31 ± 4.17 (14.91 - 27.83)	20.91 ± 4.31 (15.27 - 27.77)	0.324
	17	21	8	13	908 ± 309 (555 - 1811)	811 ± 183 (554 - 1064)	968 ± 359 (565 - 1866)	0.326	21.51 ± 4.15 (14.72 - 28.98)	21.74 ± 4.91 (14.85 - 29.17)	21.37 ± 3.81 (14.70 - 26.07)	0.71
	18	20	11	9	955 ± 227 (544 - 1289)	912 ± 255 (549 - 1293)	1009 ± 187 (544 - 1176)	0.407	21.03 ± 4.39 (14.53 - 29.10)	20.37 ± 4.34 (14.50 - 29.23)	21.83 ± 4.57 (15.00 - 26.56)	0.451
	19	20	9	11	826 ± 297 (401 - 1291)	875 ± 326 (401 - 1295)	787 ± 281 (402 - 1219)	0.451	23.27 ± 4.97 (18.47 -36.24)	21.24 ± 1.91 (18.47 - 23.60)	24.93 ± 6.10 (18.53 - 36.26)	0.094
	20-29	39	20	19	797 ± 225 (519 - 1288)	846 ± 235 (520 - 1378)	745 ± 208 (416 - 1288)	0.165	23.54 ± 5.34 (14.44 - 37.38)	22.68 ± 5.90 (12.77 - 39.68)	24.44 ± 4.67 (18.57 - 37.38)	0.407
	30-39	47	21	26	801 ± 216 (430 - 1201)	772 ± 222 (416 - 1100)	825 ± 211 (429 - 1339)	0.48	24.96 ± 6.62 (13.86 - 38.34)	23.74 ± 6.09 (12.93 - 36.76)	25.94 ± 6.98 (13.81 - 40.40)	0.32
	40-49	38	15	23	764 ± 245 (414 - 1451)	789 ± 267 (476 - 1451)	748 ± 235 (382 - 1382)	0.878	25.46 ± 6.07 (13.84 - 34.92)	23.82 ± 6.04 (13.27 - 31.97)	26.52 ± 5.97 (16.90 - 36.78)	0.262
	50-64	26	14	12	757 ± 239 (374 - 1293)	735 ± 265 (337 - 1352)	784 ± 214 (443 - 1184)	0.238	24.11 ± 7.30 (14.83 - 38.44)	27.47 ± 6.68 (15.56 - 40.58)	20.18 ± 6.09 (14.80 - 34.10)	0.09
	65-89	44	25	19	755 ± 240 (444 - 1279)	783 ± 241 (437 - 1274)	717 ± 239 (442 - 1308)	0.373	25.01 ± 6.456 (14.85 - 37.81)	24.62 ± 6.15 (14.96 - 38.04)	25.52 ± 6.97 (14.62 - 38.02)	0.516
	birth - 3d	12	6	6	1889 ± 623 (1041 - 2891)	1757 ± 570 (1126 - 2515)	2021 ± 697 (1041 - 2891)	0.675	1.18 ± 0.69 (0.31 - 2.42)	1.15 ± 0.87 (0.31 - 2.42)	1.20 ± 0.55 (0.42 - 2.02)	0.788
0-1	3d - 1m	30	15	15	3086 ± 1159 (1422 - 5223)	2786 ± 1172 (1123 - 5010)	3386 ± 1104 (1871 - 5483)	0.136	1.32 ± 0.80 (0.42 - 3.32)	1.31 ± 0.66 (0.43 - 2.70)	1.34 ± 0.94 (0.42 - 2.02)	0.783
	1m - 6m	20	11	9	2785 ± 876 (1204 - 4507)	2662 ± 578 (1867 - 3664)	2935 ± 1166 (1192 - 4529)	0.594	2.47 ± 1.26 (0.51 - 4.23)	2.55 ± 1.19 (0.50 - 4.15)	2.38 ± 1.41 (0.60 - 4.23)	0.752
	6m - 1y	19	10	9	3202 ± 1336 (2558 - 3846)	3278 ± 1329 (1368 - 4919)	3118 ± 1419 (1932 - 5776)	0.882	4.59 ± 1.42 (2.50 - 6.87)	4.38 ± 1.39 (2.50 - 6.58)	4.83 ± 1.49 (3.21 - 6.87)	0.588

CD8

	age (years)	CD8 number (/µl): total	CD8 number (/µl): male	CD8 number (/µl): female	p*	CD8 function (%): total	CD8 function (%): male	CD8 function (%): female	p*
	0	882 ± 494 (291 - 1896)	917 ± 450 (325 - 1890)	844 ± 540 (259 - 2178)	0.246	6.39 ± 4.02 (1.46 - 14.42)	6.58 ± 4.48 (1.35 - 14.83)	6.19 ± 3.50 (1.91 - 11.01)	0.972
	1	1215 ± 578 (421 - 2313)	1356 ± 741 (435 - 2366)	1101 ± 392 (406 - 2069)	0.644	18.89 ± 6.88 (8.93 - 32.11)	19.15 ± 7.30 (8.95 - 32.42)	18.67 ± 6.76 (8.90 - 31.80)	0.804
	2	956 ± 357 (477 - 1795)	978 ± 401 (476 - 1841)	917 ± 285 (645 - 1383)	0.751	20.83 ± 8.99 (9.16 - 41.53)	22.50 ± 10.47 (9.03 - 41.90)	17.91 ± 4.85 (9.90 - 24.55)	0.581
	3	836 ± 406 (377 - 1855)	942 ± 479 (369 - 1923)	683 ± 208 (433 - 1009)	0.285	23.56 ± 8.73 (11.41 - 41.79)	21.35 ± 9.06 (10.75 - 42.16)	26.75 ± 7.57 (18.42 - 39.70)	0.06
	4	881 ± 412 (232 - 1706)	806 ± 295 (231 - 1079)	971 ± 523 (306 - 1716)	0.202	27.13 ± 9.24 (15.02 - 48.93)	26.28 ± 5.93 (18.49 - 35.22)	28.15 ± 12.41 (14.91 - 49.23)	0.852
	5	770 ± 352 (267 - 1685)	877 ± 378 (437 - 1723)	692 ± 322 (176 - 1615)	0.216	27.46 ± 6.46 (13.95 - 38.82)	28.40 ± 6.55 (13.94 - 38.83)	26.77 ± 6.54 (13.97 - 38.81)	0.363
	6	783 ± 264 (331 - 1143)	787 ± 293 (287 - 1148)	779 ± 236 (486 - 1109)	0.96	28.84 ± 6.42 (15.42 - 37.60)	28.66 ± 6.75 (13.32 - 38.13)	29.06 ± 6.31 (34.44 - 36.36)	0.759
	7	810 ± 421 (285 - 1966)	676 ± 217 (287 - 1205)	989 ± 562 (285 - 1985)	0.085	26.99 ± 10.77 (10.45 - 45.84)	30.35 ± 10.49 (11.50 - 45.84)	22.50 ± 9.96 (10.43 - 36.21)	0.153
	8	728 ± 212 (429 - 1166)	799 ± 203 (558 - 1163)	682 ± 213 (408 - 1167)	0.159	28.24 ± 6.06 (19.90 - 40.05)	28.82 ± 5.91 (21.30 - 39.45)	27.87 ± 6.35 (19.70 - 40.20)	0.638
	9	784 ± 300 (426 - 1382)	789 ± 308 (482 - 1386)	779 ± 310 (423 - 1296)	0.893	28.44 ± 8.69 (10.92 - 38.13)	29.88 ± 9.02 (10.80 - 38.15)	27.00 ± 8.56 (13.26 - 37.34)	0.423
	10	619 ± 173 (367 - 920)	576 ± 162 (361 - 917)	676 ± 180 (457 - 920)	0.328	30.57 ± 8.81 (13.04 - 43.89)	31.33 ± 9.59 (12.60 - 42.00)	29.56 ± 8.10 (16.97 - 44.10)	0.683
	11	625 ± 128 (379 - 856)	630 ± 143 (376 - 818)	622 ± 125 (439 - 858)	0.639	31.96 ± 9.79 (21.03 - 55.30)	35.05 ± 11.41 (25.27 - 55.78)	30.29 ± 8.83 (21.00 - 45.92)	0.274
0-89	12	672 ± 288 (287 - 1352)	700 ± 336 (276 - 1352)	636 ± 224 (329 - 1077)	0.892	31.73 ± 8.67 (17.77 - 53.25)	33.02 ± 9.47 (17.02 - 55.15)	30.04 ± 7.66 (20.77 - 45.66)	0.278
	13	710 ± 186 (338 - 1116)	676 ± 173 (312 - 927)	749 ± 203 (582 - 1122)	0.804	32.07 ± 11.49 (16.93 - 57.34)	35.59 ± 9.92 (23.71 - 55.00)	27.84 ± 12.31 (16.80 - 57.75)	0.069
	14	600 ± 140 (356 - 787)	603 ± 113 (452 - 788)	598 ± 158 (356 - 784)	0.809	31.25 ± 9.00 (19.66 - 44.66)	32.00 ± 6.67 (21.90 - 45.00)	30.83 ± 5.80 (19.27 - 42.75)	0.758
	15	615 ± 115 (455 - 782)	591 ± 115 (457 - 780)	643 ± 115 (455 - 782)	0.323	33.62 ± 8.09 (17.69 - 45.57)	33.60 ± 8.72 (17.40 - 45.61)	33.64 ± 7.80 (20.27 - 43.97)	0.952
	16	624 ± 142 (382 - 853)	594 ± 133 (408 - 853)	649 ± 150 (381 - 849)	0.402	33.99 ± 11.46 (18.96 - 54.49)	36.47 ± 12.10 (19.96 - 54.60)	31.95 ± 11.06 (18.91 - 51.87)	0.328
	17	612 ± 257 (294 - 1066)	530 ± 152 (320 - 797)	662 ± 300 (291 - 1069)	0.575	34.00 ± 10.61 (22.23 - 56.72)	34.18 ± 10.47 (22.52 - 57.00)	33.89 ± 11.11 (22.20 - 54.15)	0.941
	18	593 ± 254 (294 - 1087)	555 ± 249 (293 - 1088)	640 ± 268 (372 - 1058)	0.451	36.44 ± 10.21 (19.04 - 54.93)	32.65 ± 8.34 (18.77 - 49.00)	41.08 ± 10.81 (24.23 - 55.17)	0.112
	19	633 ± 245 (233 - 990)	606 ± 249 (234 - 991)	655 ± 252 (233 - 967)	0.587	37.27 ± 7.06 (26.20 - 49.48)	37.40 ± 7.68 (26.13 - 49.60)	37.16 ± 6.89 (27.70 - 47.12)	0.974
	20-29	521 ± 176 (272 - 931)	567 ± 195 (274 - 970)	473 ± 144 (266 - 851)	0.119	43.78 ± 9.59 (25.98 - 60.64)	42.49 ± 9.83 (25.43 - 60.68)	45.14 ± 9.41 (25.98 - 60.64)	0.465
	30-39	521 ± 178 (290 - 867)	500 ± 151 (248 - 879)	538 ± 199 (297 - 1012)	0.63	51.31 ± 10.94 (32.64 - 70.15)	52.13 ± 9.77 (35.61 - 70.03)	50.66 ± 11.95 (29.98 - 72.33)	0.683
	40-49	433 ± 165 (259 - 828)	465 ± 231 (259 - 1058)	413 ± 105 (260 - 591)	0.889	58.26 ± 11.57 (39.28 - 77.01)	58.89 ± 12.17 (37.75 - 77.10)	57.86 ± 11.43 (40.33 - 76.96)	0.627
	50-64	357 ± 104 (194 - 569)	376 ± 106 (229 - 601)	335 ± 102 (190 - 510)	0.65	65.37 ± 10.55 (49.27 - 86.96)	66.17 ± 10.44 (53.00 - 87.74)	64.43 ± 11.06 (48.88 - 85.50)	0.441
	65-89	314 ± 157 (129 - 643)	298 ± 129 (155 - 607)	334 ± 189 (110 - 688)	0.653	75.24 ± 10.53 (56.04 - 91.40)	74.40 ± 10.40 (59.61 - 90.60)	76.33 ± 10.90 (54.81 - 94.90)	0.586
	birth - 3d	539 ± 186 (256 - 847)	595 ± 181 (313 - 847)	484 ± 189 (256 - 704)	0.387	4.22 ± 1.78 (1.35 - 7.23)	3.92 ± 1.63 (1.35 - 5.41)	4.51 ± 2.01 (1.42 - 7.23)	0.788
0-1	3d - 1m	919 ± 370 (366 - 1603)	893 ± 341 (288 - 1480)	945 ± 407 (434 - 1622)	0.86	3.98 ± 1.75 (1.44 - 7.43)	3.75 ± 1.63 (1.00 - 6.40)	4.22 ± 1.89 (1.91 - 8.39)	0.56
	1m - 6m	732 ± 330 (264 - 1444)	796 ± 368 (392 - 1456)	653 ± 277 (259 - 1096)	0.402	6.57 ± 2.92 (1.44 -11.84)	6.52 ± 3.12 (1.37 - 11.88)	6.64 ± 2.85 (3.47 - 11.01)	0.974
	6m - 1y	1197 ± 719 (360 - 2757)	1277 ± 585 (548 - 2004)	1109 ± 872 (360 - 2757)	0.287	11.36 ± 4.12 (6.77 - 21.64)	12.48 ± 4.28 (6.77 - 21.64)	10.12 ± 3.79 (6.87 - 19.68)	0.112

NK

	age (years)	NK number (/µl): total	NK number (/µl): male	NK number (/µl): female	p*	NK function (%): total	NK function (%): male	NK function (%): female	p*
	0	519 ± 235 (270 - 985)	530 ± 251 (305 - 1038)	507 ± 219 (216 - 935)	0.657	68.51 ± 12.56 (44.73 - 87.91)	67.59 ± 14.94 (42.48 - 90.03)	69.50 ± 9.45 (53.41 - 85.71)	0.695
	1	482 ± 195 (230 - 825)	473 ± 189 (257 - 857)	489 ± 206 (202 - 793)	0.799	68.62 ± 12.08 (46.68 - 87.42)	67.29 ± 11.65 (49.15 - 82.76)	69.70 ± 12.68 (44.20 - 90.14)	0.296
	2	513 ± 237 (221 - 980)	517 ± 226 (216 - 978)	506 ± 272 (247 - 980)	0.789	74.81 ± 12.56 (46.43 - 88.63)	72.67 ± 14.52 (45.56 - 88.80)	78.57 ± 7.54 (64.14 - 87.65)	0.566
	3	475 ± 235 (214 - 1019)	509 ± 265 (238 - 1057)	427 ± 189 (212 - 704)	0.383	71.74 ± 10.79 (45.01 - 87.68)	72.29 ± 9.475 (59.20 - 88.10)	70.93 ± 13.02 (42.51 - 85.32)	0.835
	4	464 ± 181 (264 - 799)	489 ± 196 (264 - 809)	435 ± 166 (264 - 735)	0.595	77.77 ± 7.28 (58.38 - 88.46)	78.33 ± 8.65 (56.30 - 88.55)	77.10 ± 5.60 (70.16 - 83.88)	0.447
	5	414 ± 179 (228 - 808)	413 ± 145 (276 - 749)	415 ± 205 (227 - 840)	0.47	75.68 ± 10.51 (54.68 - 91.60)	78.36 ± 10.48 (54.70 - 91.60)	73.71 ± 10.44 (54.67 - 91.59)	0.279
	6	418 ± 196 (216 - 890)	430 ± 203 (211 - 879)	403 ± 195 (227 - 895)	0.875	78.97 ± 9.37 (61.54 - 93.53)	76.24 ± 10.42 (61.26 - 91.59)	82.45 ± 6.79 (71.21 - 94.36)	0.133
	7	438 ± 146 (202 - 728)	454 ± 140 (278 - 738)	418 ± 160 (199 - 635)	0.663	75.41 ± 9.77 (61.31 - 95.29)	77.54 ± 10.19 (61.21 - 96.00)	72.56 ± 8.95 (62.25 - 88.86)	0.242
	8	468 ± 173 (180 - 785)	486 ± 167 (242 - 736)	456 ± 181 (165 - 797)	0.682	79.72 ± 11.91 (52.42 - 93.31)	77.81 ± 13.76 (50.40 - 91.10)	80.95 ± 10.92 (60.49 - 93.50)	0.669
	9	435 ± 162 (216 - 726)	442 ± 141 (237 - 719)	428 ± 189 (215 - 726)	0.8	77.94 ± 9.45 (58.84 - 90.70)	78.24 ± 10.21 (59.40 - 90.70)	77.65 ± 9.16 (58.81 - 90.39)	0.836
	10	428 ± 167 (238 - 782)	417 ± 154 (254 - 637)	442 ± 192 (236 - 798)	0.745	81.31 ± 6.12 (64.61 - 91.55)	81.31 ± 7.40 (63.25 - 91.57)	81.30 ± 4.30 (76.82 - 90.59)	0.542
	11	459 ± 208 (202 - 965)	526 ± 247 (256 - 977)	423 ± 184 (200 - 720)	0.392	78.94 ± 9.02 (53.64 - 90.99)	80.07 ± 12.43 (53.00 - 91.04)	78.33 ± 7.10 (65.71 - 90.09)	0.241
0-89	12	388 ± 143 (214 - 648)	385 ± 151 (222 - 661)	391 ± 140 (212 - 560)	0.867	77.27 ± 9.53 (58.24 - 93.44)	76.45 ± 9.01 (60.92 - 91.37)	78.32 ± 10.56 (57.57 - 93.96)	0.685
	13	423 ± 171 (213 - 740)	420 ± 164 (212 - 728)	426 ± 188 (218 - 741)	0.902	78.73 ± 8.01 (63.67 - 90.78)	79.76 ± 6.71 (67.74 - 89.90)	77.50 ± 9.57 (63.56 - 90.93)	0.614
	14	345 ± 147 (173 - 609)	347 ± 166 (173 - 613)	343 ± 141 (174 - 584)	0.943	77.07 ± 9.33 (59.09 - 89.15)	81.10 ± 9.91 (59.60 - 89.28)	74.77 ± 8.48 (59.00 - 86.13)	0.059
	15	369 ± 148 (164 - 659)	371 ± 132 (230 - 661)	367 ± 172 (164 - 645)	0.844	80.35 ± 6.59 (66.74 - 90.13)	81.15 ± 6.64 (70.20 - 90.20)	79.46 ± 6.78 (66.36 - 88.61)	0.418
	16	373 ± 201 (156 - 849)	411 ± 217 (170 - 854)	342 ± 192 (156 - 760)	0.407	80.64 ± 6.90 (60.37 - 89.96)	81.88 ± 9.09 (59.75 - 89.98)	79.63 ± 4.67 (72.17 - 89.59)	0.095
	17	351 ± 140 (149 - 604)	351 ± 141 (212 - 610)	350 ± 145 (147 - 549)	0.962	82.21 ± 6.50 (70.29 - 93.18)	80.93 ± 6.22 (69.80 - 90.68)	83.01 ± 6.79 (74.65 - 93.36)	0.71
	18	313 ± 177 (83 - 666)	333 ± 187 (90 - 673)	289 ± 172 (83 - 493)	0.589	79.13 ± 8.20 (61.36 - 94.12)	79.13 ± 8.87 (62.40 - 94.37)	79.13 ± 7.83 (61.30 - 88.41)	0.755
	19	325 ± 175 (79 - 629)	348 ± 181 (78 - 612)	306 ± 177 (102 - 630)	0.538	83.21 ± 7.17 (67.95 - 91.52)	81.73 ± 8.05 (68.61 - 91.53)	84.41 ± 6.51 (67.91 - 91.30)	0.444
	20-29	401 ± 216 (123 - 872)	488 ± 214 (199 - 1042)	310 ± 182 (119 - 691)	0.006	80.60 ± 9.24 (62.11 - 94.60)	80.21 ± 10.30 (61.74 - 96.69)	81.01 ± 8.24 (62.11 - 92.89)	0.828
	30-39	374 ± 191 (168 - 832)	449 ± 225 (197 - 989)	313 ± 134 (147 - 624)	0.023	82.49 ± 7.79 (66.65 - 93.26)	83.79 ± 8.62 (64.69 - 93.44)	81.44 ± 7.05 (68.17 - 92.69)	0.162
	40-49	367 ± 186 (119 - 756)	484 ± 226 (156 - 960)	291 ± 102 (104 - 450)	0.004	81.83 ± 8.38 (66.00 - 93.86)	81.85 ± 10.17 (64.08 - 95.12)	81.81 ± 7.22 (67.86 - 93.00)	0.911
	50-64	476 ± 264 (177 - 1053)	584 ± 280 (176 - 1072)	350 ± 184 (179 - 759)	0.035	82.47 ± 8.24 (62.99 - 95.03)	84.13 ± 8.33 (70.34 - 95.29)	80.53 ± 8.03 (59.03 - 89.00)	0.55
	65-89	609 ± 296 (120 - 1151)	722 ± 295 (140 - 1185)	459 ± 226 (104 - 891)	0.003	80.55 ± 9.68 (61.45 - 94.67)	81.19 ± 9.78 (57.89 - 94.57)	79.71 ± 9.75 (60.00 - 97.30)	0.952
	birth - 3d	402 ± 128 (305 - 685)	377 ± 107 (305 - 588)	428 ± 152 (317 - 685)	0.513	68.94 ± 13.04 (44.54 - 84.67)	67.01 ± 16.38 (44.54 - 84.67)	70.88 ± 9.83 (53.41 - 81.61)	0.898
0-1	3d - 1m	436 ± 187 (217 - 870)	404 ± 146 (305 - 775)	468 ± 221 (216 - 987)	0.675	68.99 ± 14.06 (43.24 - 90.79)	65.57 ± 16.27 (42.20 - 91.47)	72.41 ± 10.94 (50.60 - 86.15)	0.215
	1m - 6m	506 ± 230 (219 - 962)	561 ± 254 (312 - 963)	439 ± 190 (215 - 705)	0.146	68.24 ± 11.91 (36.66 - 83.55)	66.11 ± 14.42 (36.15 - 83.60)	70.83 ± 7.93 (61.00 - 80.85)	0.7
	6m - 1y	737 ± 228 (371 - 1087)	776 ± 257 (371 - 1087)	693 ± 198 (416 - 935)	0.412	67.76 ± 11.32 (54.31 - 88.87)	72.58 ± 13.71 (54.31 - 88.87)	62.40 ± 3.95 (58.66 - 70.02)	0.128

d, day; m, month; y, year. *Comparisons were performed between male and female for the number and function of $CD4^+$, $CD8^+$ T cells and NK cells using Mann–Whitney *U* test. Data were presented as means ± SD (5th−95th centiles).
